# Ponseti Technique for the Management of Congenital Talipes Equinovarus in a Rural Set-Up in India: Experience of 356 Patients

**DOI:** 10.3390/children5040049

**Published:** 2018-04-10

**Authors:** Rohit Malhotra, Ashutosh Mohapatra, Geetika Arora, Priyam Choudhury, Hitesh Joshi, Pranav Patel

**Affiliations:** 1 Department of Orthopaedics, ESIC Model Hospital, Baddi 173205, Himachal Pradesh, India; dr.rohit.malhotra@esic.in; 2 Department of Orthopaedics, Mohapatra Hospital, Ulhasnagar 421003, Maharashtra, India; 3 Department of Anaesthesia, PGIMER, Chandigarh 160012, India; geetika.arora3010@gmail.com; 4 MBBS, Dr DY Patil Medical College and Hospital, Pimpri 411018, Maharashtra, India; priyamchoudhury28@gmail.com; 5 Department of Orthopaedics, RDBP Jaipuria Hospital, Jaipur 302018, Rajasthan, India; hitesh192@yahoo.co.in; 6 Departmentof Orthopaedics, Pimprikar Hospital, Nasik 422009, Maharashtra, India; patel-mail@pimprikarhospital.com

**Keywords:** congenital talipes equinovarus, clubfoot, Ponseti, Pirani score, rural set-up, India

## Abstract

Congenital talipes equinovarus (CTEV), also known as clubfoot, is a complex congenital deformity of the foot that, left untreated, can limit a person’s mobility by making it difficult and painful to walk. Worldwide, 80% of children born with clubfoot are in low- and middle-income countries. The management of clubfoot has a long history. Non-operative management did not become popular, as an increasing number of orthopaedists started leaning towards surgical treatment. The late Dr. Ignacio Ponseti developed a method of clubfoot correction that successfully realigns clubfoot in infants without extensive and major surgery. The aim of the study was to assess the functional outcome of CTEV management by the Ponseti technique, to study the severity of CTEV deformity using the Pirani score, and to evaluate the cost-effectiveness of the technique. A total of 356 cases with 402 feet with CTEV were treated by the Ponseti method. The average age of the children and the number of casts applied before full correction were 4.03 months and 6.91, respectively. There was a good functional outcome in 95.45% of cases (score > 30) at the last follow up. The management of CTEV by the Ponseti technique provides a good functional and cosmetic outcome. In a developing country like India, this technique is a safe, easy, economical method of clubfoot management.

## 1. Introduction

Congenital talipes equinovarus (CTEV), also known as clubfoot, is a complex, congenital deformity of the foot. When untreated, children with clubfoot walk on the sides and/or tops of their feet, resulting in callus formation, potential skin and bone infections, inability to wear standard shoes, and substantial limitations in mobility and employment opportunities [1]. The incidence of congenital clubfoot is approximately 1 in every 1000 live births. It has a male predominance of 2:1 and an incidence of bilateralness estimated to be about 50% [2]. According to the Global Clubfoot Initiative report, the incidence of children born with clubfoot in India is 30,000 per year [3].

Worldwide, 80% of children born with clubfoot are in low- and middle-income countries (LMICs) [4]. A large proportion of these remain untreated or poorly treated, leaving them to face a life of disability. A neglected clubfoot causes crushing physical, social, psychological and financial burdens on the patients, their families and society [5]. Many of these cases are untreated or poorly treated, leading to a neglected clubfoot. These children undergo extensive corrective surgery, often with disturbing failures and complications. Revision surgeries are also, thus, more common. Although the foot looks better after surgery, it is stiff, weak and often painful. After adolescence, the pain increases and often becomes crippling [6]. There is nearly universal agreement that the initial treatment of clubfoot should be non-operative, regardless of the severity of the deformity. The late Dr. Ignacio Ponseti developed a method of clubfoot correction by manipulation and casting, based on the fundamentals of kinematics and pathoanatomy of the deformity, which successfully realigns clubfoot in infants without extensive and major surgery [7]. The Ponseti method has a success rate of up to 92–100% worldwide, with surgical rates decreasing by 7% per year after peaking in 2000–2001, and only 10% of cases requiring surgical intervention beyond a tenotomy to achieve a good functional outcome [8]. Over the years, there have been a number of scoring systems described for clubfoot. These include the Ponseti-Laaveg classification, the Dimeglio classification, etc. These are quite cumbersome to use and have not proved popular. In order to assess the level of severity of each of the components of clubfoot effectively, Shafique Pirani MD designed a convenient and easy tool known as the Pirani Score [9]. Widely implemented in high-income countries, the Ponseti method has been described as highly suitable for healthcare settings with scarce resources and is being increasingly used in low- and medium-income countries as well [10,11].

The present study was undertaken to assess the functional outcomes of CTEV management by the Ponseti technique, to study the severity of CTEV deformity using the Pirani score, and to assess the cost-effectiveness of the Ponseti technique in a rural set-up in a developing country like India.

## 2. Materials and Methods

### 2.1. Study Area

This was a prospective clinical study done in the orthopaedic and paediatrics department of a rural primary health centre in the state of Maharashtra, India, between 2013 and 2017. A total of 379 patients were selected based on inclusion and exclusion criteria. The parents of 13 patients initially agreed for the treatment but, after the whole study protocol was explained to them, backed out of the trial citing reasons such as inconvenience to the patients and duration of the study. This can be attributed to the fact that our study consisted of subjects coming from very low-income backgrounds like farmers, labourers, drivers and industrial workers who are illiterate and uneducated, and hence did not understand the importance and benefit of the procedure for their children. Surgical intervention was offered to all of the 13 patients, with four willing to undergo it while the remaining nine shunned it totally. In spite of our best efforts to explain the Ponseti procedure to them repeatedly, they were unwilling to proceed and hence these 13 patients were excluded from the study. The remaining 366 patients formed our study group. We lost 10 patients in follow up due to reasons like relocation of the patients, inconvenience to the subjects due to Ponseti casts, and transportation costs incurred by the parents. Hence, the final study consisted of 356 subjects with 402 CTEV feet.

### 2.2. Ethical Considerations

The clearance from the ethical committee (DY Patil Hospital, Pimpri research committee) was obtained before the start of study with the project code: IEC 0051/2013 dated 13/07/2013. The informed, written consent of each participant was obtained from the parents prior to the study by the issuance of a consent form.

All the subjects of the study were treated by the Ponseti method. Parents were educated about the condition, management technique and, more importantly, the course of the Ponseti method.

### 2.3. Inclusion and Exclusion Criteria

All patients aged <12 months with normal hips and spine having clubfoot, and with consent to participate, were included in the study. Patients of children >12 months of age having associated neurological defects, spine and hip conditions, and previously treated by other methods, were excluded from the study.

According to the Ponseti classification, children aged less than 2 years are considered as untreated clubfoot [12,13]. A thorough search of literature revealed that there have been many published studies done on children up to 2 years of age, but there have been few studies consisting of children for up to 1 year of age [14,15,16,17,18,19,20]. Hence, we decided to conduct our study on children aged ≤12 months. The age groups were divided as (a) ≤4 months (b) >4–8 months and (c) >8–12 months in order to provide a comprehensive and detailed review of the Ponseti method, as no study has been having a detailed age demarcation except one [20] has been previously undertaken.

A complete history of the patient’s condition was taken from the parents which included any other associated anomalies, any family history of the same condition or a history of consanguious marriage among the parents, and maternal obstetric history.

All patients were evaluated in detail about laterality, sex distribution, and severity of deformity. A general examination was undertaken to rule out any other congenital anomalies.

### 2.4. Evaluation of Final Outcome

The severity of deformity and the functional outcome was graded according to the Pirani scoring system both at the beginning and at the end of the treatment. This detects the degree of correction. It scores according to 6 clinical signs: 3 for mid-foot, 3 for hind-foot. Three signs of mid-foot score (MS) and hind-foot score (HS) grade the amount of deformity between 0 and 3. The Pirani score 0 means a normal foot, score 3 means a moderately abnormal foot, score 6 means a severely abnormal foot [21]. We graded our results as excellent, good and poor with 0 to 0.4: excellent, 0.5 to 1: good and >1: poor. Excellent and good results were a reflection of the success of the treatment while the poor results were deemed as failures and offered surgical treatment.

Extensive counseling was given to the parents with regards to the plan and method of treatment, the duration of the treatment, the postoperative protocol, the importance of braces and compliance with them, and the goal of Ponseti method.

The treatment was done in 2 stages (Figure 1, Figure 2, Figure 3, Figure 4, Figure 5, Figure 6, Figure 7 and Figure 8):

(a) correction of the deformity by weekly serial casting;
(b) maintenance of that correction by bracing.

Casting was begun as soon as possible when the children came to us for treatment. In all the infants, the Pirani scoring was done to assess the initial severity. Weekly follow up were undertaken during the initial periods of bracing to ensure compliance and to periodically assure and educate the parents. Later, after the application of a Dennis Brown (DB) splint, a monthly follow up was advised for three months and then up to 4 years.

### 2.5. Statistical Analysis

Data analysis was undertaken using Statistical Package for the Social Science (SPSS) Version 17 [22] for Windows. The demographic variables and other variables were calculated by number and percentage. The Wilcoxon test was used to find a significant difference in Pirani score and functional rating before and after treatment, and a chi-square test was used to find an association between sex and laterality. Correlation and a test for correlation was used to find the significant correlation between the Pirani score and the total number of casts. A probability value of 0.05 was accepted as the level of statistical significance.

## 3. Results

### 3.1. Age

In our study, there were 180 males and 176 females. The mean age at presentation was 4.03 months, and the age-range from 0.5 to 12 months. The most common age group at presentation was ≤4 months i.e. 225 cases (63.33%); 83 cases (23.33%) were between the ages of >4–8 months; and 48 cases (13.33%) were more than 8 months and less than 12 months of age (Table 1).

### 3.2. Laterality

Among the 356 cases, 166 cases (46.67%) had bilateral clubfoot, and 190 cases (53.33%) had unilateral clubfoot out of which 106 (56%) were right-sided and 84 (44%) were left-sided.

### 3.3. Consaguinity and Family History

We found that 153 cases (43.33%) were born out of consanguineous marriages of parents and 203 cases (56.33%) had a positive family history of clubfoot.

### 3.4. Pirani Score

**Figure 9:**
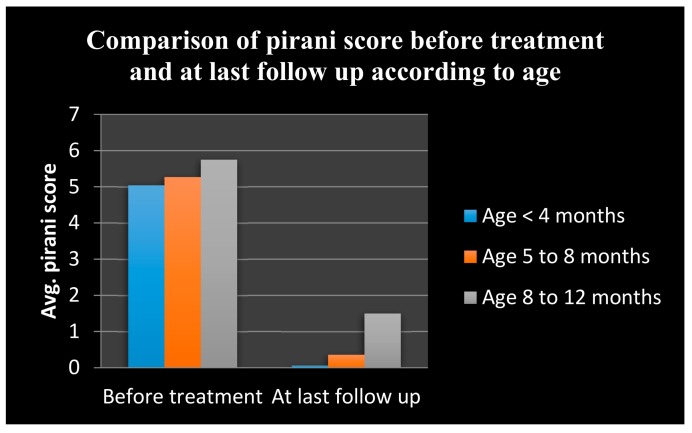
Bar diagram for Pirani score distribution.

In our series, the mean initial Pirani score at the time of presentation of cases was 5.19 (range 3.5 to 6) and at last follow up was 0.33 (range 0 to 3.5) (Table 2 and Figure 9).

- The mean + standard deviation (SD) at initial and final Pirani score in the age group of ≤4 months were 5.04 + 0. 85 and 0.06 + 0.21, respectively.
- The mean + SD at initial and final Pirani score in the age group of >4–8 months were 5.27 + 0.65 and 0.36 + 0.39, respectively.
- The mean + SD at initial and final Pirani score in the age group of >8–12 months were 5.75 + 0.61 and 1.50 + 1.41 respectively.

### 3.5. Number of Casts

The mean total number of casts required to correct the deformity was found to be 6.91, ranging from 5 to 10 (Table 3). In the age group of ≤4 months, the mean + SD total number of casts required was 6.56 + 0.75. In the age group of >4–8 months, the mean + SD total no. of casts required was 7 + 1. In the age group of >8 to 12 months, the mean + SD total number of casts required was 8.33 + 1.86.

### 3.6. Complications

**Figure 10:**
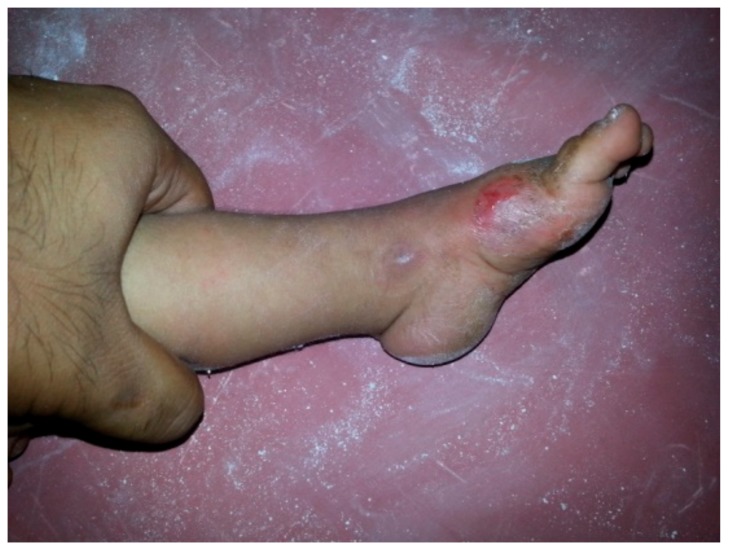
Skin abrasions.

**Figure 11:**
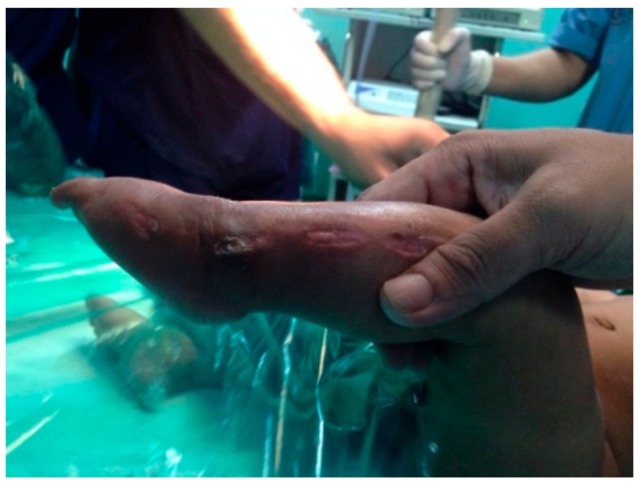
Cast saw injury.

In our series, a few minor complications were encountered during the casting procedure which included skin abrasions, cast saw injuries, cast loosening and cast breakage (Figure 10 and Figure 11).

### 3.7. Final Outcome

Based on the Pirani score, the final functional outcomes were graded as excellent, good and poor and consisted of 275, 41 and 40 patients, respectively (Table 4).

### 3.8. Comparison of Cost of Treatment

Total charges that we incurred for each patient were 71 USD, which was comparatively less than for the Turcos procedure charges of 96 USD and hence very affordable for each of the patients (Table 5).

## 4. Discussion

CTEV is one of the commonest congenital deformities. It is a complex deformity comprising equinus, varus, adductus and cavus, which are difficult to correct. It requires a meticulous and dedicated effort on the part of the treating physician and parents for the correction of the deformity The goal of treatment is to reduce or eliminate these deformities so that the patient has a functional, pain-free plantigrade foot with good mobility without calluses and does not need to wear modified shoes [15]. India is the second most-populous country in the world, with 25% of its people (about 375 million) living below the poverty line. Approximately 25,000 children are estimated to be born with idiopathic clubfoot every year in India. With such a large population living in poverty, non-invasive treatment of clubfoot with the Ponseti method has the potential to make a large impact on health outcomes for children who would otherwise be crippled by it [23]. Our study aims to evaluate the Ponseti method by using the Pirani score as a functional tool and to measure the cost-effectiveness of the Ponseti method.

### 4.1. Demographic and Etiological Variables

In our study, the average age of presentation was 4.03 months, with the most common age at presentation being ≤4 months i.e. 225 cases (63.33%), and with 131 cases (36.67%) presenting between >4 months–12 months (Table 1), which is comparable to M. Changulani et al. who treated 66 patients with 100 idiopathic clubfeet using the Ponseti method and reported the mean age at presentation of 12 weeks or three months (1 to 60 weeks) [24]. Our study had 180 cases (63.33%) in males and 176 (36.67%) in females, with a male to female ratio of 1.02, which is similar to the study of M. Changulani et al. who found that 50 patients (75.75%) were males and 16 patients (24.24%) were females [24]. As regards laterality, 166 cases (46.67%) had bilateral clubfoot and 190 cases (53.33%) had unilateral clubfoot (106 right-sided and 84 left-sided) which accords with other studies done by Ponseti et al., Changulani et al., Lehman et al., Christian et al. and Pavone et al. [7,24,25,26,27] (Table 6).

In our study, 153 cases (43.33%) had a history of consanguinous marriages of parents as compared to 203 cases (56.67%) who did not, which is comparable to the study by T. Sreenivas and A.R. Nataraj, which had, 54 (31%) out of 174 born of a consanguinous marriage [28]. This suggests a probable role of consanguinity as an etiological factor in the development of CTEV. Our study included 117 cases (33.33%) with a positive family history of clubfoot, which is similar to Morcuende et al. who evaluated 157 patients and reported that 22% of them had a positive family history of clubfoot deformity [6].

### 4.2. Pirani Score

The above findings signify that as the age of presentation increases the severity of the deformity increases. A comparison between the mean initial Pirani score and mean Pirani score at the last follow up shows that the Ponseti method was effective in treating the deformity in all the age groups of our study, as the *p*-value remains statistically significant in all age groups, although highly significant in the lower age group of <4 months (<0.0001) when compared with the higher age group >4–8 months (<0.005) and >8–12 months (<0.05). This clearly implies that the earlier the treatment begins, the better are the results. The available literature suggests that the results were better if this method of treatment was started as early as possible after birth [29,30].

### 4.3. Tenotomy and Number of Casts

In our study, 309 feet (77%) required tenotomy to correct the equinus deformity whereas 47 feet (23%) required only casting to the equinus deformity, which is comparable to studies by Changulani et al., Lehman et al., and Christian S. et al., Pavone et al. [24,25,26,27] (Table 7).

The mean total number of casts that we applied up to the final follow up to correct the deformity was 6.9 (ranged from 5 to 10) which was comparable to similar studies done by Changulani et al., Lehman et al., Christian et al. [24,25,26] (Table 8).

### 4.4. Complications

We encountered 4 types of complications among all the castings performed, which included the following: 57 cases (16%) had complications out of which 10 cases had skin abrasions, 36 cases had a cast breakage, 2 cases had a cast saw injury, and 9 cases had cast loosening and slippage. There were no major complications such as infection, skin necrosis, neurovascular compromise or profuse bleeding after tendoachilles tenotomy. Lehman et al. [25] reported having a 10.2% complication rate. The complications included cast saw injuries, abrasions, cast intolerence, maceration, blisters and slough.

### 4.5. Final Functional Outcome

We used the Pirani score as a functional tool for the assessment of the functional outcome. The results were graded as excellent, good and poor. Out of the 356 patients treated, excellent results were achieved in 275 cases (77.2%) while good and poor results were evenly distributed with 41 cases (11.2%) and 40 cases (11.1%), which is comparable to Sakale H et al. which had 92% excellent results [31]. Out of the 225 patients in the age group of ≤4 months, excellent results were achieved in 220 patients while two patients presented with poor results. These two patients did not follow the bracing protocol properly. The age group of >8–12 months had the maximum number of failures with 28 patients out of 48. This futher reinforced the belief that treatment begun earlier provides the best results. These 28 patients were offered surgery in the form of Turco’s procedure; 12 parents refused surgery citing the cost of the procedure as the main reason.

### 4.6. Cost-Effectiveness

Total charges that we incurred on each patient amounted to 71 USD, which was comparatively less than for the Turcos procedure charges of 96 USD and very affordable for the patients’ families (Table 6). Our findings in this regard were similar to the studies by Gadhok et al., Ferreira et al. and Hussain et al. [22,32,33]. Since our study group consisted of patients coming from low-income backgrounds in rural areas, cost was a very important variable for the success of the treatment.Grimes CT et al. [34] in their study also stated that the Ponseti method for the treatment of club foot is cost-effective and practical in a low-income country setting. The rural population in India was reported at 66.86% (% of total population) in 2016, according to World Bank development indicators [35], which makes the Ponseti method arguably the best for countering the menace of clubfoot.

## 5. Conclusions

Clubfoot or CTEV can be successfully graded by the Pirani score which is reproducible and does not show any inter-observer variation. This scoring system includes all the parameters in evaluating a clubfoot deformity and helps in decision-making as to whether to continue the ongoing management or opt for a surgical intervention. The Pirani scoring system also helps in documenting the progress of treatment and evaluating its results.

Management of CTEV by the Ponseti technique provides good functional and cosmetic outcomes when treated with strict adherence to the guidelines given by Ponseti that include serial casting, maintenance by bracing, and parental education, which can be started as soon as the child is born.

In a developing country like India, where there is a scarcity of resources and the referral system is poor, the Ponseti method is a very safe, efficient, cost-effective, economical treatment for the correction of clubfoot that radically decreases the need for extensive corrective surgery while also decreasing the social and financial burden of the parents, which can also be used successfully in rural contexts in other developing countries.

## Figures and Tables

**Figure 1:**
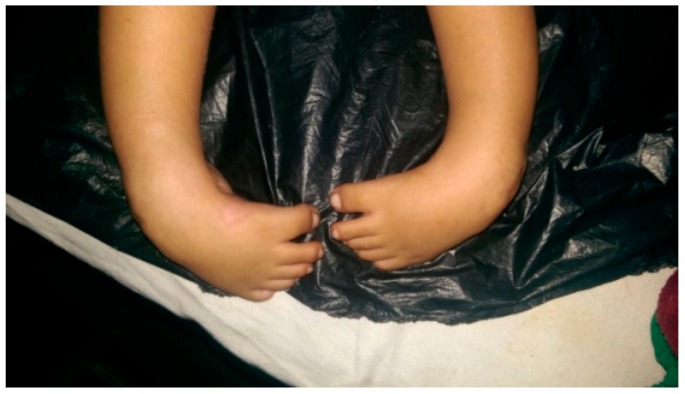
Initial presentation.

**Figure 2:**
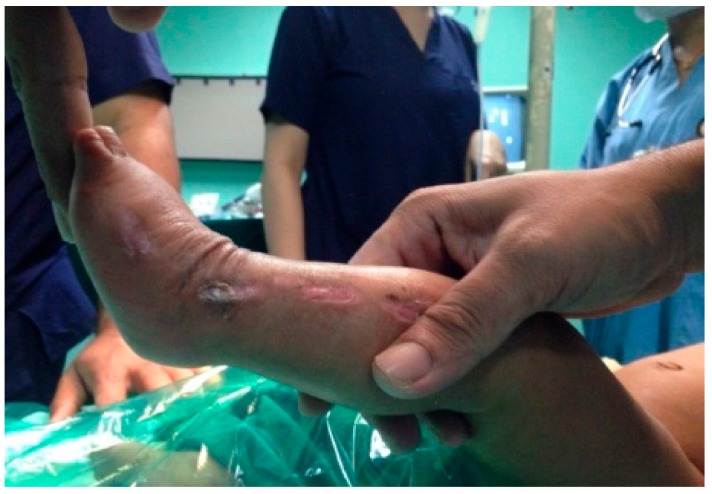
Manipulation.

**Figure 3:**
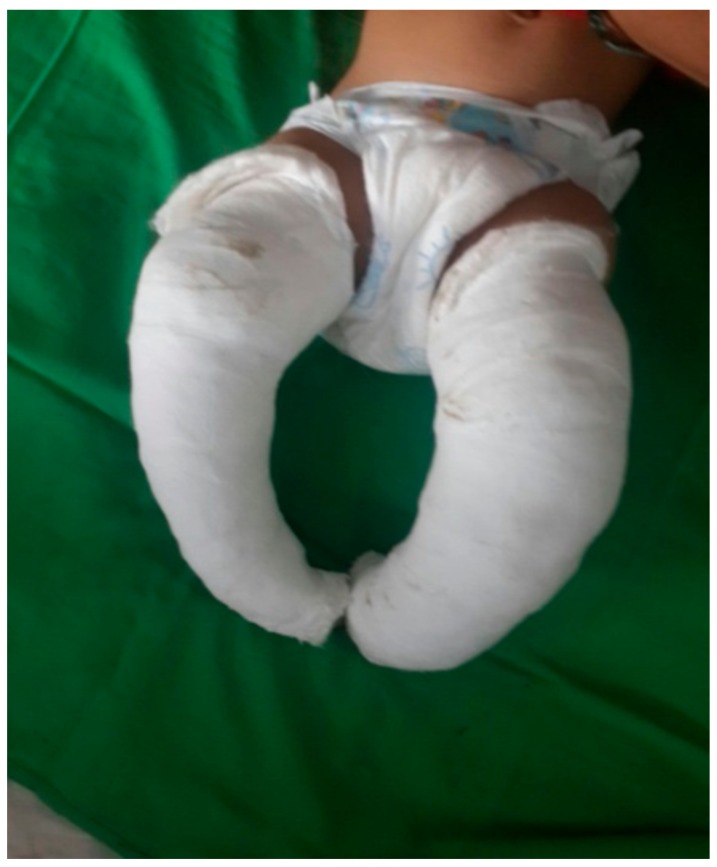
First cast for cavus correction.

**Figure 4:**
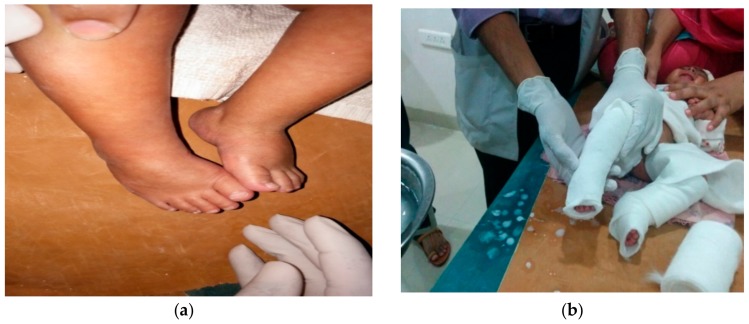

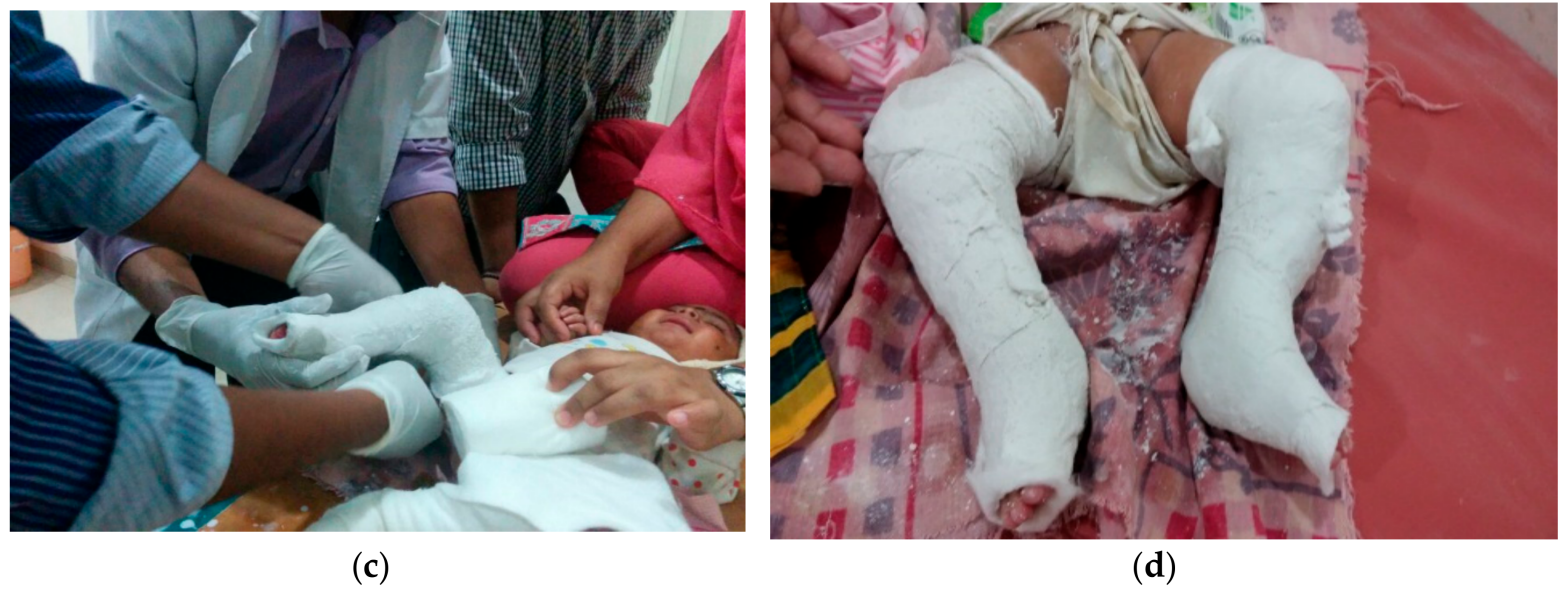
(**a**–**d**) Subsequent steps in manipulation and casting (casts 2–4).

**Figure 5:**
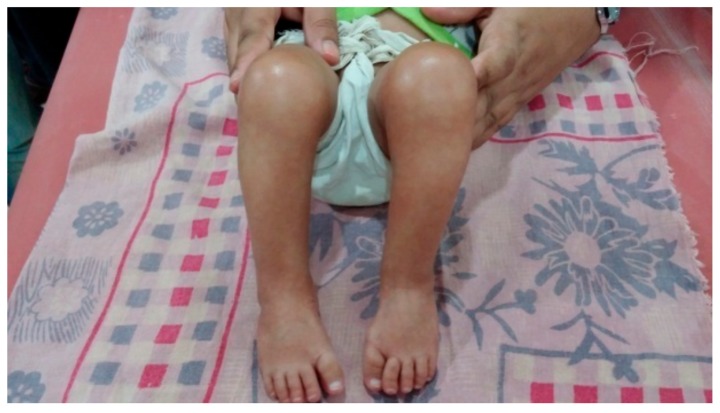
After removal of fourth cast.

**Figure 6:**
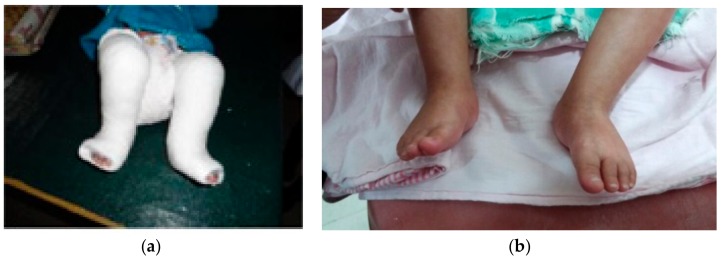
(**a**) Last cast (fifth cast) for equinus; (**b**) after removal of fifth cast.

**Figure 7:**
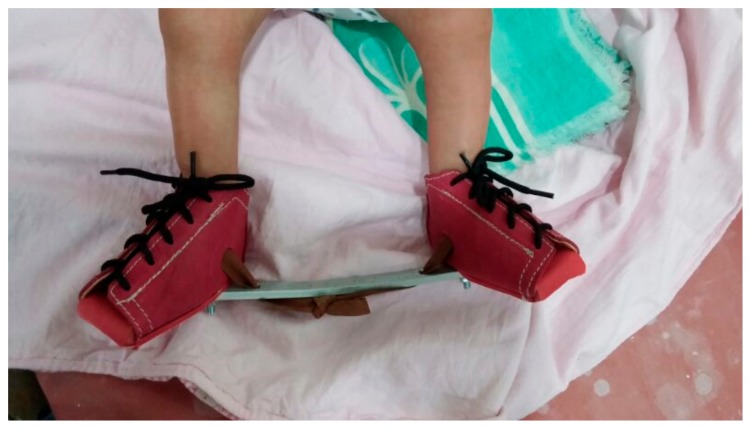
Dennis Brown (DB) splint and shoes.

**Figure 8:**
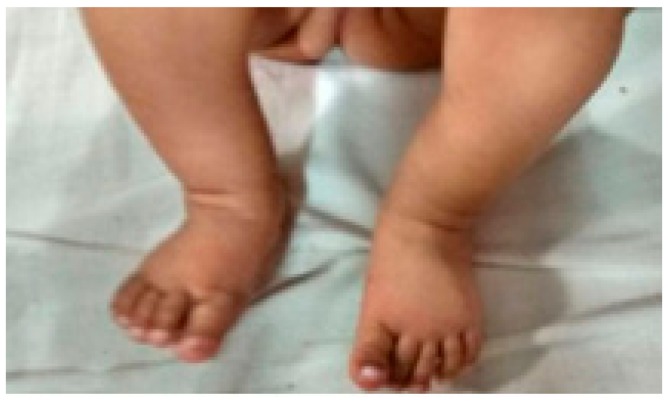
Clinical picture at last follow up.

**Table 1:** Age distribution of patients.

Age of Patients (Months)	Number of Patients	Percentage (%)
≤4	225	63.33
>4–8	83	23.33
>8–12	48	13.33
Total	356	100

**Table 2:** Pirani score distribution pre- and post-treatment.

Age (Months)	Pirani Score (Pre Treatment)		Pirani Score (Last Follow Up)		Wilcoxon *Z* Value	*p* Value
	Mean	SD	Mean	SD		
≤4	5.04	0.85	0.06	0.21	5.61	<0.0001
>4–8	5.27	0.65	0.36	0.39	2.99	<0.005
>8–12	5.75	0.61	1.50	1.41	2.21	<0.05

SD: Standard deviation

**Table 3:** Age distribution of casts.

Age (Months)	Total Number of Casts	
	Mean	SD
≤4	6.56	0.75
>4–8	7	1
>8–12	8.33	1.86

**Table 4:** Distribution of final outcome.

Age (Months)		Functional Outcome	
	Excellent	Good	Poor
≤4	220 (97.77%)	03 (1.3%)	02 (0.8%)
>4–8	53 (63.9%)	20 (24%)	10 (12%)
>8–12	02 (4%)	18 (3.7%)	28 (58.33%)
Total	275 (77.2%)	41 (11.5%)	40 (11.2%)

**Table 5:** Distribution of cost.

Ponseti Method (Variables)	USD	Turcos Method (Variables)	USD
Cast and other consummables	25	Pre-operative antibiotics	06
Tenotomy charges	11	Pre-operative investigations	12
Dennis Brown (DB) Splint	19	Removal of stitches and k wires	14
Other hospital charges	16	Post-operative antibiotics	12
		Hospital stay (3 days)	26
		DB splint and ankle foot orthosis	26
Total	71		96

**Table 6:** Comparison of laterality with other studies.

Studies	Unilateral (%)	Bilateral (%)
Ponseti et al. [7]	40 (60%)	27 (40%)
Lehman et al. [25]	15 (50%)	15 (50%)
Changulani et al. [24]	32 (48%)	34 (52%)
Christian et al. [26]	70 (60%)	46 (40%)
Pavone et al. [27]	50 (61%)	32 (39%)
Our study	190 (53.33%)	166 (46.67%)

**Table 7:** Comparison of tenotomy with other studies.

Studies	Percentage of Feet Requiring Tenotomy
Changulani et al. [24]	85% of feet
Pavone et al. [27]	72% of feet
Lehman et al. [25]	75% feet
Christian S. et al. [26]	79% of feet
Our Study	77% of feet

**Table 8:** Comparison of casts applied with other studies.

Studies	Casts
Lehman et al. [25]	5.4 (range 4 to 9)
Changulani et al. [24]	6 (range 2 to 12)
Christian S. et al. [26]	7.2 (range 3 to 13)
Our Study	6.9 (range 5 to 10)
